# Some Cases in Cattle-practice1Read before the December meeting of the Missouri Valley Veterinary Medical Association.

**Published:** 1898-03

**Authors:** R. H. Harrison

**Affiliations:** Atchison, Kan.


### SOME CASES IN CATTLE-PRACTICE.[1]

SELECTED BY R. H. HARRISON, D.V.S.,
ATCHISON, KAN.

RUMENOTOMY. A thoroughbred Jersey cow, five years of age, in an extremely emaciated condition.

*History.* Came of healthy parents, and was, up to her fourth year, the mother of two healthy heifer-calves. She lost her third calf the fourth month after conception; at this time had no serious trouble, but shortly after went dry, and was not bred again for eight months. Two months after aborting she began to lose condition, although generously fed and nursed; her appetite was very capricious, and at times was noticed to cough, especially on getting up, and to eat foreign bodies, and at times was affected with diarrhœa, which would last a week or ten days. At no time was any discharge noticed from the vulva. When called, the owner said that she seemed especially fond of rusty iron, and he thought she had swallowed an old iron currycomb that had been broken and thrown in the cow-lot; he knew she had swallowed the handle, but was not sure of the rest of it, and he thought she was choked "low down."

When seen, the animal was lying down naturally in a loose box; extreme emaciation; abdomen tucked up, flanks very hollow; on getting up, coughed spasmodically for a full minute. Temperature subnormal, 95°; respiration 8; pulse 32; very weak; staggers when made to walk; nose dry; mucous membranes very pale; coat dry and staring, and shows the remains of the greater part of winter's coat. Pressure over the larynx provokes no cough, but exercise in walking does. Examination, per vaginam, shows os uteri closed; per rectum, outlines an empty uterus; udder normal. Examination of the lungs shows resonance over all the chest, except on the left side; low down between the fifth and ninth ribs, a hand's-breadth square, dulness detected; on auscultation, the respiratory murmur was very weak on both sides of chest, and least over dull portion. Fetid diarrhœa present; no food had been taken for thirty-six hours; but two gallons of corn and oatmeal-gruel, with raw eggs, had been given by drenching. On making

[1] Read before the December meeting of the Missouri Valley Veterinary Medical Association.

pressure over left flank, pushing rumen forward, the animal shows distress, and coughs. The operation of rumenotomy was advised, and the animal prepared by feeding, or rather giving, punches of eggs, grape-brandy and milk several times a day for a week, as well as one ounce of Liebig extract in solution, daily.

The operation consisted in opening the rumen and removing the currycomb in two pieces, four pounds of nails, five pounds of broken spikes, a dress skirt, parts of several boots, shoes and rubbers ; a coil of baling-wire ; twenty-four balls of hair, the largest the size of an orange, the smallest the size of a walnut ; a cog-wheel, 1½ inches in diameter ; pieces of wood, and much clay. There was very little food present. In examining the cavity of the rumen, by means of portable electric light, discovered a piece of wire penetrating the rumen into the chest ; its extraction gave rise to much pain, and was followed by an escape of two quarts of creamy pus, with a slight hemorrhage. A small sound was introduced through this opening for a distance of twelve inches, but its end could not be felt on the outside of the chest. The abscess was washed out with diluted $H_2O_2$, and the rumen sponged with a 2 per cent. solution of creolin, and a gallon of cooked chopped hay, oatmeal, and cornmeal was introduced into the rumen. The incision of the rumen was closed by turning edges in, using kangaroo-tendon suture, made continuous ; the opening of the flank was closed with quill sutures and reinforced by a bandage ; animal was given punches, as before, in generous quantities, and continued for forty-eight hours, when some mashed carrots and apples, with steamed clover-hay, were taken naturally. Twenty-four hours after operation the temperature was .101°, but animal looked brighter ; wounds appeared to be doing well. Forty-eight hours after, temperature normal. Begins to eat, and the punches are given less frequently ; drinking-water is given often, in small quantities (wound of flank doing well), in which is placed fl. ext. nux vomica and gentian, together with sulphite soda and salicylic acid ; *no iron.* The stitches in wound of flank are removed in a week, which is cicatrized in two weeks ; bandage is still kept in place with a pad of creolated oakum over wound.

*Result.* Animal recovers health, strength, and condition ; gives birth to a healthy calf, and dies two years after the operation, from tympanitis, as a result of low choking.

*Post-mortem,* made four days after death : find body well nourished, and the usual lesions of tympanitis, together with rupture of walls of rumen ; in thoracic cavity find a closed cyst, filled with a little serum, between pleuræ and adhesions of pleural surface to lung; the rest of the alimentary tract was carefully examined, but no foreign bodies found; the cause of choking was a whole potato.

This is the first case in my practice among bovines where I used animal food, and where food was introduced into the stomach from the outside. It was done as an experiment, for I did not expect the patient to recover.

FRACTURE OF PENIS. *Subject.* A five-year old Durham bull.

*History.* Was kept in a pasture with twelve cows and eight yearling and two-year old heifers. Was observed to cover a cow Saturday and Sunday; to repeatedly attempt to cover a heifer ineffectually. Monday evening came up with the cows and showed much distress, making repeated attempts to pass urine, straining violently, bellowing and pawing. The owner noticed the penis protruding several inches from the prepuce, covered with dirt, and so swollen that it could not be forced back into place. He said he took a shingle and scraped the dirt off, and that it bled very freely. The next morning, the animal being down and unable to get up, he returned the organ within the prepuce, after greasing it, and noticed a small quantity of urine voided after much straining.

On my visit, the next day, found animal down beside some straw ricks, in agony, with a strong ammoniacal odor from the breath and body; bellowing, groaning, and straining so violently that the rectum was prolapsed; had lost all control over the hind legs, but would pull himself along with his forelegs. Was unable to expose penis on account of a large swelling within the prepuce. Examination through inverted rectum showed bladder enormously distended. On pressure, and the animal straining violently, a pint of very high-colored and strong-smelling urine is evacuated in a very small stream; this manipulation gave rise to an agony of pain, and the animal died immediately.

*Post-mortem:* At once. Bladder the size of a bushel basket, with walls thickened and mucous lining congested; urine high-colored and ammoniacal; ureters and pelvis of kidneys filled with urine; kidneys congested; incising prepuce and exposing penis shows a dark-colored swelling four inches from meatus, the size of a man's fist, which on incision shows a bloody tumor, with a complete disintegration of cavernous tissue, the spaces being one cavity and the partitions as merely débris; the extremity of the organ is excoriated, ulcerated, and but slightly swollen. This is, I think, a rare case, and one that could properly be called a fracture; an organ with its meshes (cavernous tissue) filled with blood, in a state of erection, to receive a sudden violent blow, or bending, sufficient to cause a breaking or fracture of tissue.

It occured to me that possibly, by earlier attention, the patient might have been saved by relieving the distention of bladder by puncture or perineal incision and by treating the fractured organ afterward.

EPITHELIOMA OF CLITORIS. Holstein cow, seven years old.

Presents a pear-shaped tumor, hanging from within the vulva, involving the clitoris, excoriated, bleeding very easily, and very erectile. Belonged to an ignorant German farmer, and was kept on account of her milking quality. Tumor had been growing for a year, and the owner had used a powder composed of burnt-alum and powdered sulphate of copper over it, with no result; the animal was a very bad kicker, and it was necessary to tie her hind-legs together every time she was milked. She had not been bred for two years; came in rut regularly, and was especially vicious, and the tumor very erectile and sensitive during the period.

*Operation.* The tumor and the clitoris were removed in three days by the application of a strong elastic ligature, renewed daily, the parts being kept clean with creolin solution. The advantages of this method of extirpation, which was more successful and advantageous than the knife or cautery, were, first, that it was bloodless, and, again, that the secondary slough following ligation by elastic ligature is of much benefit, for you can get rid of the root of a malignant growth more certainly than by other means.

*Result.* The case did well; the parts were kept clean, after sloughing had taken place, until cicatrized with a 10 per cent. solution of creolin. Animal was bred in due time, and up to three months ago, eighteen months after the operation, had had no return of the tumor.

CALCULI, PREPUTIAL AND URETHRAL. A Durham yearling bull. No previous history; was noticed to strain violently, and pass no urine; was caught up and examined by owner, an intelligent stockman, who found the tuft of hair at end of prepuce matted together in such a manner as to close the opening completely; this was softened with warm water, and an unsuccessful attempt made to draw the penis out.

Was called immediately and found animal in much distress; constant tenesmus, quickened respiration, sweating, and looking around at left flank and groin. Pulsations could be seen and felt at the perineum; the prepuce shows a mass of sebaceous and sabulous matter filling its opening and extending under the penis a distance of two inches. A rectal injection of a pint of linseed oil and one-half ounce of chloroform was given, which relieved the tenesmus. The state of the bladder could not be determined on account of the anus being too small to admit the hand. The mass filling prepuce was readily removed, having been softened by the owner washing and injecting with hot water. In trying to force penis out at prepuce it was grasped above the S in the groin, where a foreign body was felt, filling the urethra; pressure of this enlargement caused exquisite pain and violent straining. Hoping to reach and dislodge the calculus, and explore the bladder at the same time (the rectum not admitting the hand), a linear incision was made in the perineum, into the urethra just below the anus, allowing a great quantity of urine to escape, which was high-colored, but contained no sabulous matter. Examination of the bladder with steel sounds show its cavity free from calculi. The attempt made to remove calculus found below, through this opening, failing on account of the very small size of the urethra, an incision was made directly over the stone, and it was readily removed; it was the shape and size of a large bean, studded with rugosities, and weighed six drachms; it had become sacculated just above the curve, or S, in the penis at the groin; this wound was made *clean*, and interrupted sutures taken, reinforced with collodion and iodoform. The prepuce was cleaned with hot creolin solution, and a mild astringent, to be injected once daily, was left. The wound in perineum was left open for twenty-four hours, with a catheter introduced into the bladder, and afterward closed with a stitch. The animal was placed on rations of grass in small quantities, and a cupful of flaxseed-jelly used in every bucket of drinking-water, and quiet ordered.

*Result.* The case did very well; wound over calculus healed by first intention; the perineal wound, which was irritated by catheter, was three weeks in cicatrizing. In operating again, and having but a perineal wound, I would suture at once, and reinforce stitches, as there is but very little areolar tissue between the urethra and skin at this point.

This animal was sold to a relation of the owner, who lives in Nebraska, and up to the present time, a year after the operation, no ill reports have been sent.

Rabies. A grade Durham cow, five years old, belonging to the same owner as the bull with fracture of penis. Had acted strangely for a few days, showing great irritability, desiring to fight other stock; has fallen off in milk and flesh; has no appetite, but takes usual amount of water. Has made vicious attacks on the owner and his helper. A bulldog belonging to the farmer had left suddenly two weeks before the cow showed the first uneasiness; the dog and this particular cow were great enemies, and she had been bitten on the nose, hocks, shins, and tail. Before calling me the owner had separated her from the rest of the herd, and had chained her up in a basement stable, as she was so vicous with his hens, ducks, and young pigs when loose in the corral.

When seen the animal appeared very alert; eyes bright and protruding, would make a vicious lunge at you as you stood in front of her, and when a harmless old pig was shown she had a paroxysm of fury, climbing into the manger with her forefeet, bellowing very hoarsely, and frothing at the mouth; leading another cow in front of her caused another frantic attempt at attack. Allowed to quiet down for a time, and watched through a window twenty feet away, noticed she would make vicious rushes at chickens and hens that would come near; would attack imaginary objects, butting post and manger so viciously that both horns and cores were fractured close to the head, and a considerable hemorrhage was set up. She presented a pitiable object—face covered with blood, hemorrhage from both nostrils, frothy and stringy saliva in abundance from the mouth, and bellowing constantly; temperature 100°, pulse and respiration accelerated; passage of feces and urine suspended.

*Diagnosis.* Rabies, and advised destroying the animal, but owner insisted on keeping her until she died, as he had given a dose of oil before my attention, and during this manipulation, which was difficult, had gotten some of the saliva on his hands, which were covered with many scratches. He was very much frightened, but I tried to allay his fear, giving him a strong creolin solution with which to wash his hands, and cauterized the wounds with nitrate of silver and brought him to town to see a physician as a safeguard to himself. I telephoned the doctor, after my client left me at my office, the nature of the case, and told him to reassure the man, as I had done. With some saliva collected from the cow I inoculated a dog, and had a well-developed case of dumb rabies in twenty days.

Allow me to call your attention to a point worthy of our consideration : As professional and honorable men, we should in similar cases endeavor to let our self-conceit and what aplomb we may gain from newspapers, their readers, and our clients be nothing in comparison to our consideration for the welfare of our client, whose mind and body might be influenced and affected with fear of this dread disease.

Ascites Amnion. Jersey cow, four years old ; undersized. Had gone a month over time, according to the record of the owner, but nothing abnormal had been noticed. Was called at night to see her, as she had been "trying to calve for several hours." Found animal standing up, with an enormously distended abdomen ; distention seemed equal on both sides, giving the animal a peculiar appearance, her length being equal to her breadth. As the pains were infrequent and the os closed, and the animal comfortable, left her for the night. The next morning the pains were more frequent, but the animal's strength was not so good as the night before ; the cervix was still rigidly closed. Examination per rectum can outline a uterus enormously distended, but no fœtus detected. After great difficulty the cervix and os were opened somewhat, and the "bag of waters" filled opening at once ; giving myself and the animal a chance to rest a little, she lay down, and very strong labor-pains began, with no result ; examining the vagina again, find cavity of vagina nearly filled with membranes ; being unable to rupture the sac with my fingers, opened it with a bistoury, and was deluged with amniotic fluid. I think it a low estimate to say that a full barrel of fluid escaped. After the escape of this fluid the patient seemed very much exhausted, and was given a pint of whiskey and an ounce of aromatic spirits of ammonia. Examination brought on very feeble pains, but no fœtus could be detected. Allowed to rest again for an hour, and one-half ounce doses of fluid extract of ergot given every half hour, which brought on the contractions vigorously, the animal showing much uneasiness, getting up and down. This time felt a foot downward and backward above the udder, which was secured and given to an assistant, who, making traction, another leg was secured, and a dead fœtus, the size of a small rabbit, extracted. As the placenta was very adherent and the animal very weak, did not attempt to remove it ; left stimulants to be given every hour, and gave a grave prognosis. When seen again, twelve hours later, had just expired.

*Post-mortem.* Body very greatly emaciated, a most striking contrast to her appearance when first seen; the membranes were thickened and very adherent to the cotyledons of the uterus, which was enlarged, with walls thin and flabby, with its fundus directed downward and backward. In this case, I must confess, that I diagnosed twins, and I can assure you that I was somewhat annoyed to show to the owner, instead of twins, a bit of a calf no larger than a small rabbit.

